# Supplementary material for: Cdh2, a downstream target of Hes7, regulates somitogenesis by supporting FGF signalling
Source: Development. 2025 Sep 15;152(17):dev204743. doi: 10.1242/dev.204743 (PMC12516320; doi:10.1242/dev.204743)
Supplement: Supplementary information [file develop-152-204743-s1.pdf]

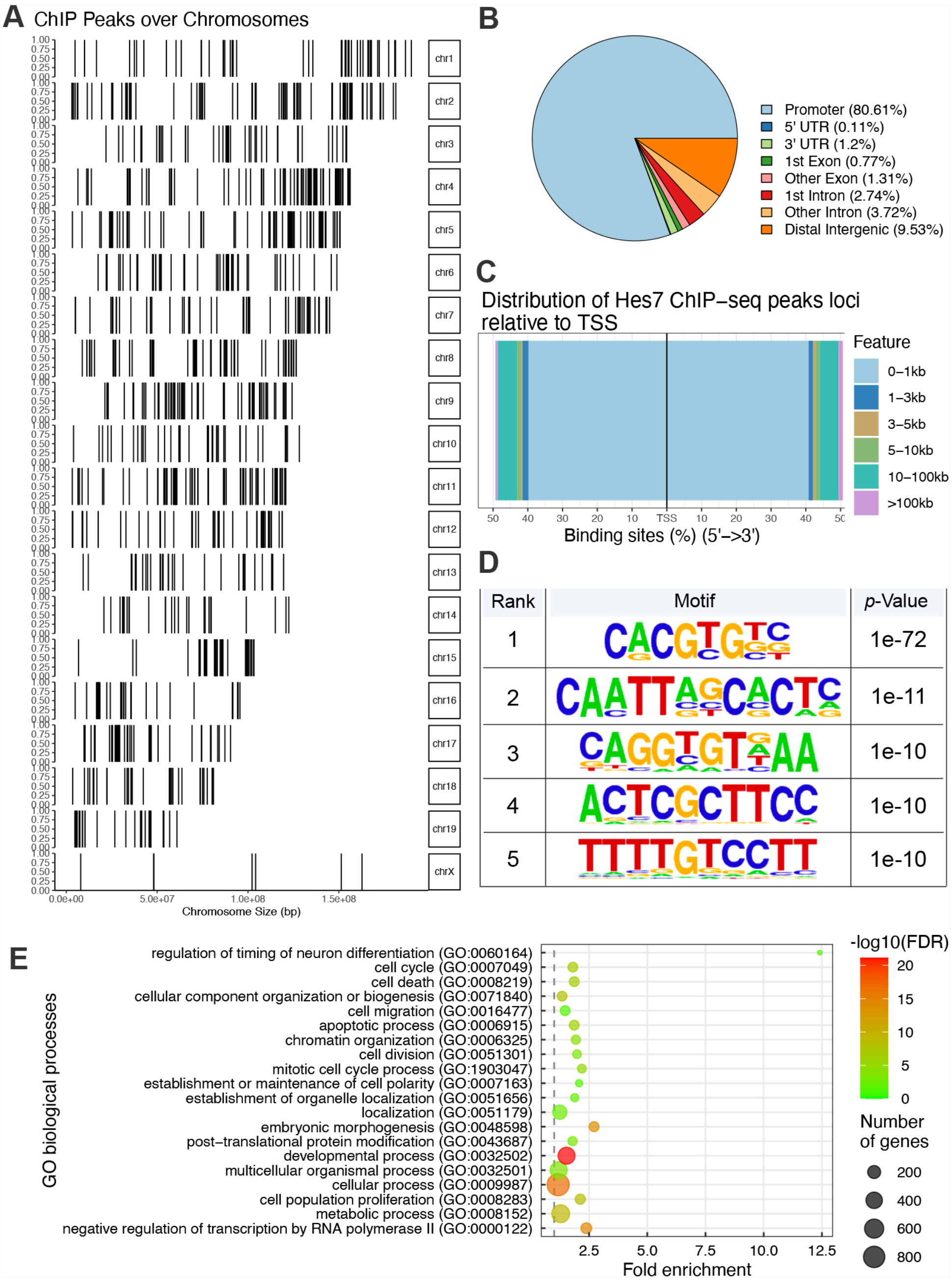

Fig. S1. Identification of genome-wide Hes7 binding sites by ChIP-seq.
(A) The distribution of reads on chromosomes from ChIP-seq data. (B) Pie diagram showing the genomic occupancy of Hes7 binding sites as revealed by ChIP-seq in the PSM. (C) Bar diagram showing the percentage of Hes7 binding sites upstream and downstream from the transcription start site (TSS) of the nearest gene. (D) Top five predicted Hes7 binding motifs with the most significant *p*-values. (E) Top 20 enriched Gene Ontology (GO) terms of the Hes7 targets.

Rank	Gene	Hes7 ChIP-seq binding	Phenotype	GO term
2	Scarf2	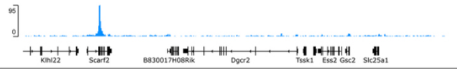	NC	Cell-cell adhesion
4	Smoc1	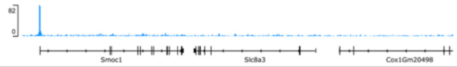	NC	extracellular matrix organization
6	Lfng	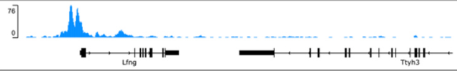	Desynchro-nization	Notch signalling pathway
9	Hes7	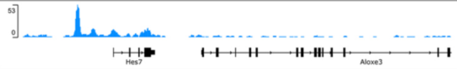	Sustained expression	Negative regulation of transcription by RNA polymerase II
12	Irf2bp2	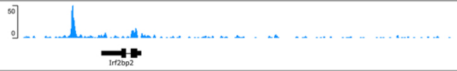	NC	Negative regulation of transcription by RNA polymerase II
13	Kpnb1	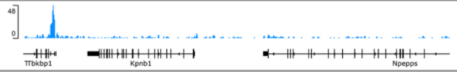	ND	Protein import into nucleus
17	Tmem131l	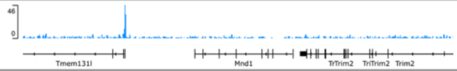	NC	Negative regulation of canonical Wnt signaling pathway
19	Khdrbs1	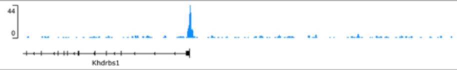	NC	mRNA processing
26	Trim71	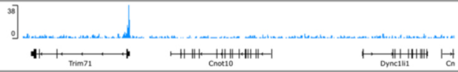	NC	miRNA processing
37	Foxo3	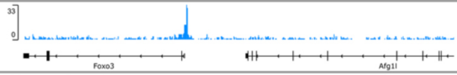	NC	Regulation of transcription by RNA polymerase II
40	Jak3	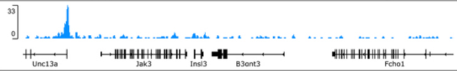	NC	Intracellular signal transduction
46	Cdh2	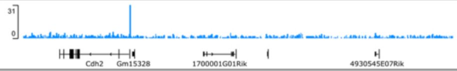	Dampened	Cell migration
53	Bcl2l11	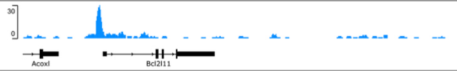	NC	Programmed cell death
61	Pcbp1	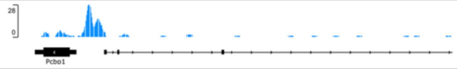	NC	Positive regulation of transcription by RNA polymerase II
79	Ier5	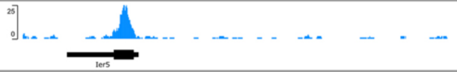	NC	Positive regulation of transcription by RNA polymerase II
88	Rab34	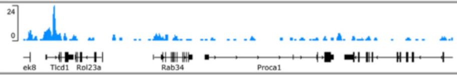	NC	Endocytosis
142	Eno3	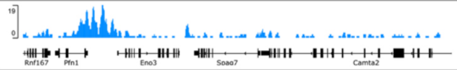	NC	Glycolytic process
482	Tead4	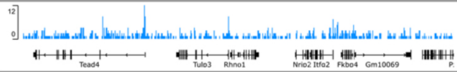	NC	Hippo signaling
611	Cited	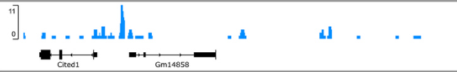	NC	Positive regulation of transcription by RNA polymerase II
639	Tra2b	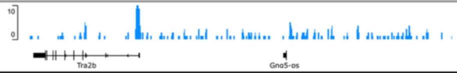	Cell apoptosis	Regulation of RNA splicing

Fig. S2. ChIP-seq profiles for genes selected for knockout screening.
The Hes7 binding peaks in mouse E10.5 PSM are visualized using the online IGV Browser. Peak enrichments are shown in blue, and the gene schematic is shown below in black. KO phenotype and GO term of each gene are indicated. NC: no change; ND: no data.

Development • Supplementary information

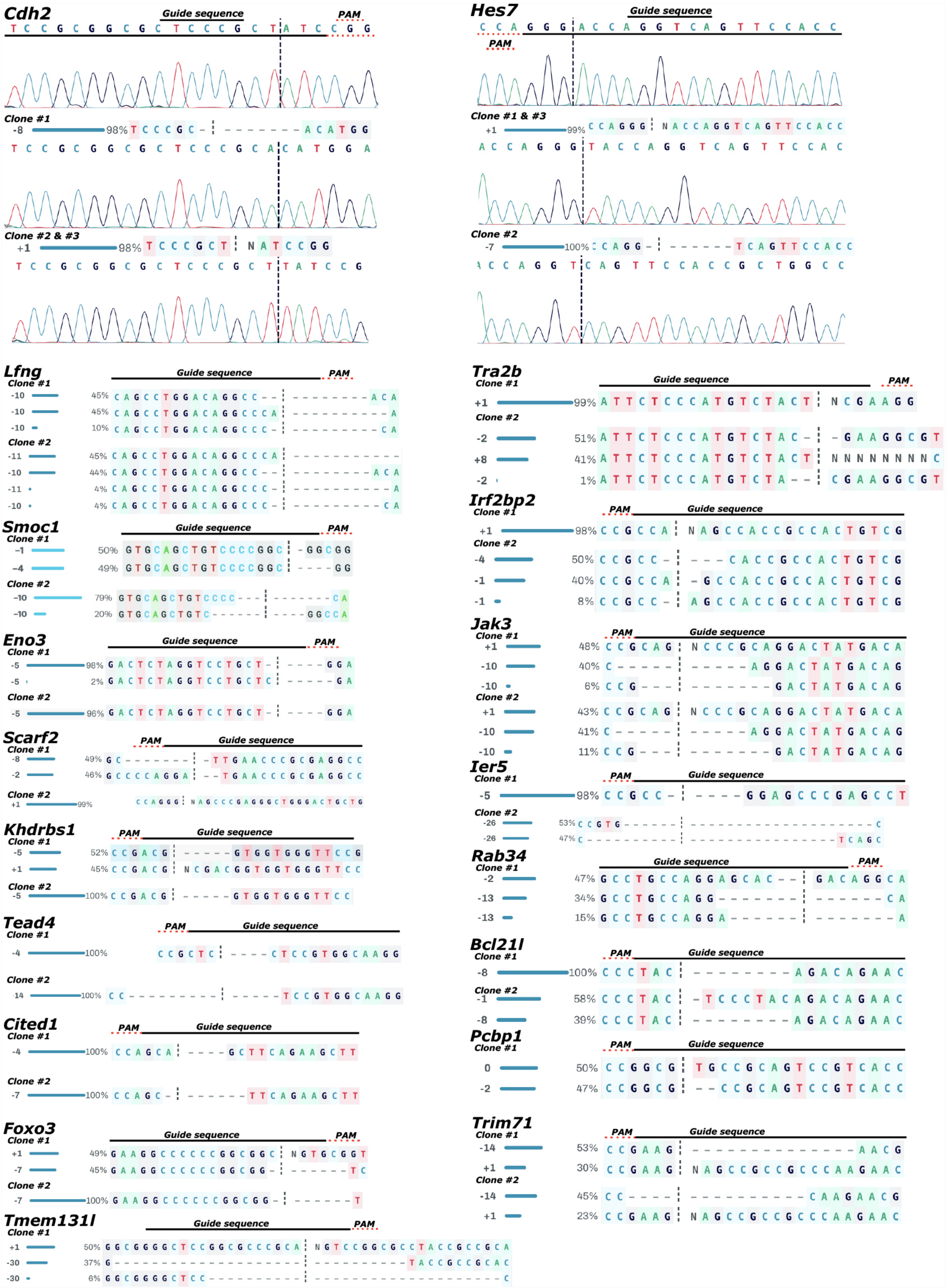

Fig. S3. Genotype sequencing of selected genes for KO screening. KO mutants generated by gene editing were sequenced.

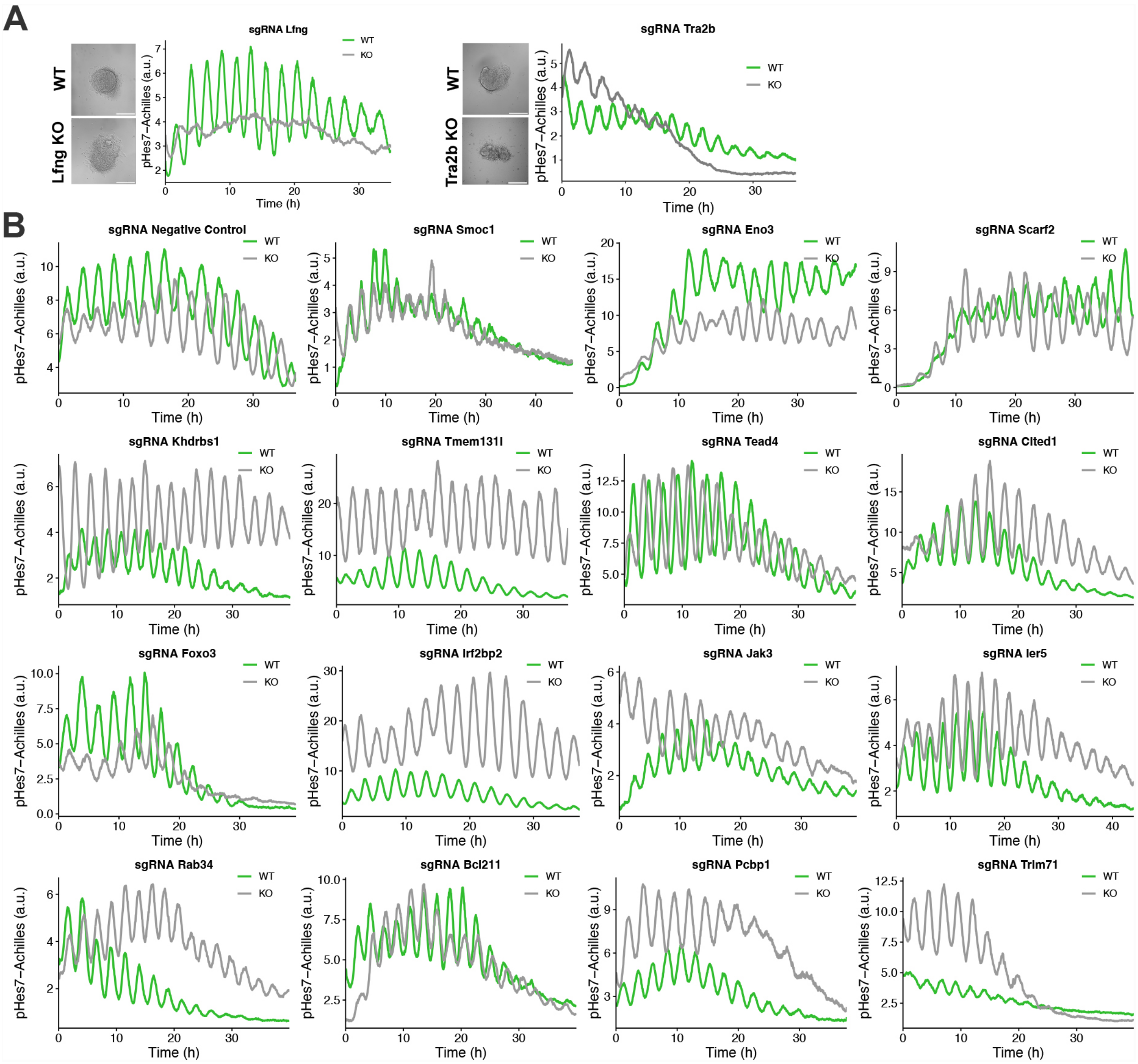

Fig. S4. Knockout screening of genes selected from the ChIP-seq binding list. Time series plots of pHes7-Achilles activity in iPSM tissue from 99 h to approximately 140 h showing a phenotype (A) and no phenotype (B). For each gene, three clones were tested and showed similar results. Only representative data are presented. a.u., arbitrary units.

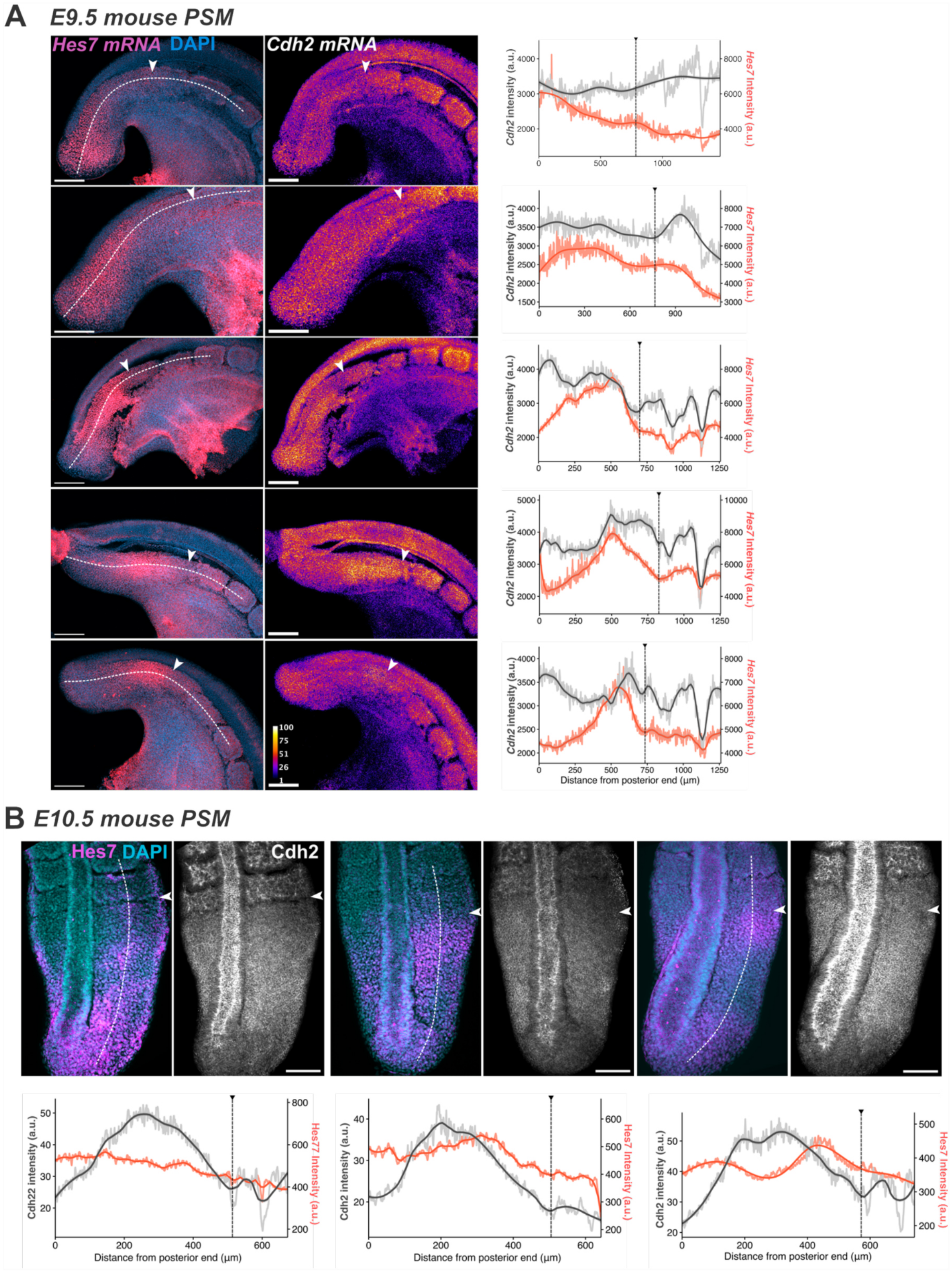

Fig. S5. Cdh2 mRNA and protein expression in mouse PSM

(A) Left: Representative hybridization chain reaction staining images to examine *Cdh2* mRNA (fire) and *Hes7* mRNA (red) expression patterns in E10.5 mouse PSM. Right: Intensity profile was plotted along the indicated lines shown in the left panels. (B) Top: Representative immunofluorescent images of Cdh2 protein (grey) and Hes7 protein (red) expression patterns in E10.5 mouse PSM. Bottom: Intensity was plotted along the indicated lines shown in the top left panels. a.u., arbitrary units. The vertical lines with arrowheads indicate the boundary between the PSM and somites, which correspond to the white arrow heads in the images. 0 indicates the posterior end. Images are the average intensity projections of 20 µm z-stacks. Scale bars, 100 µm.

Development • Supplementary information

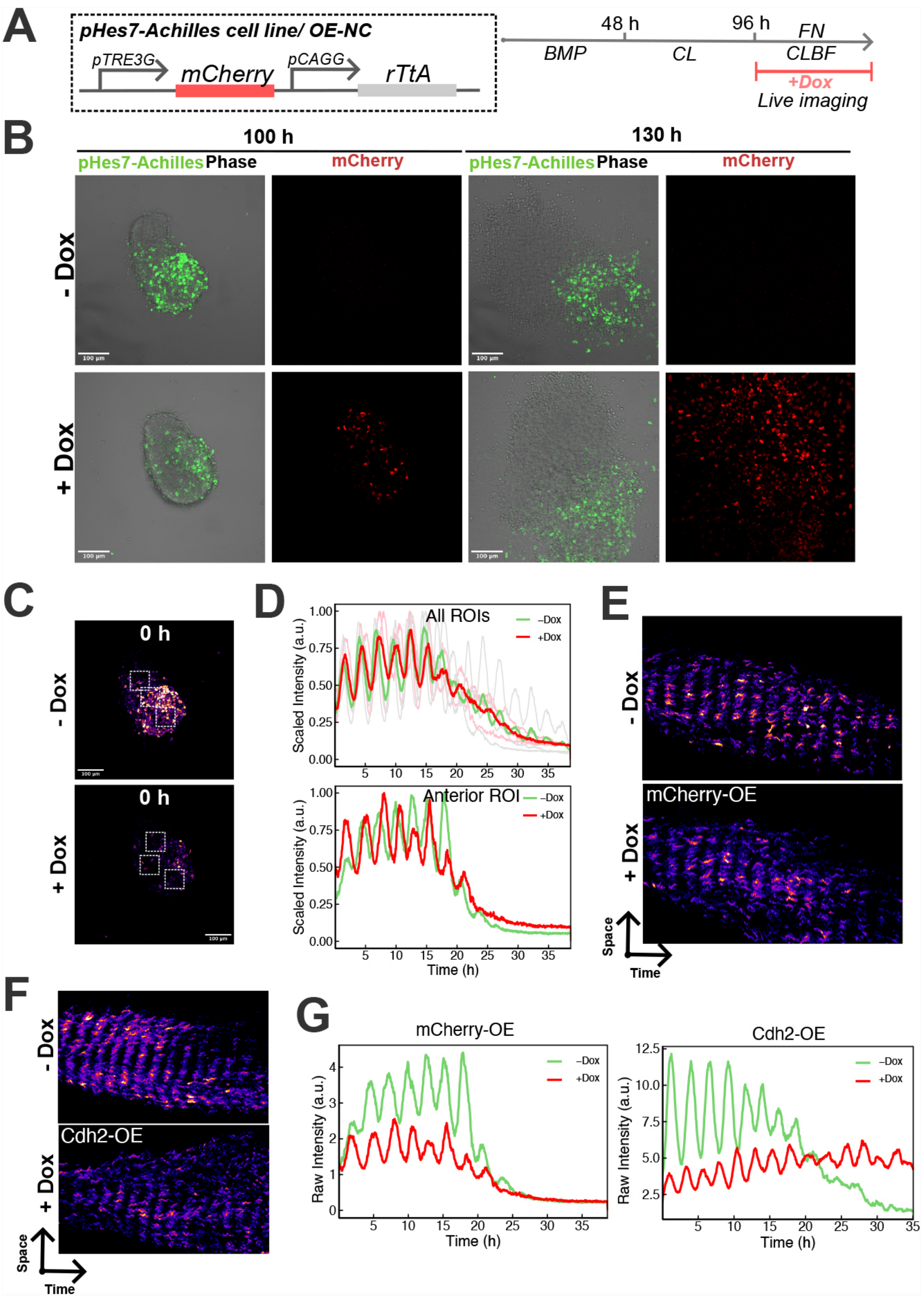

Fig. S6. Overexpression of mCherry in iPSM tissues did not affect Hes7 oscillations. (A) Scheme of the overexpression construct (left) and iPSM induction protocol (right) (B) Representative snapshots of Achilles and mCherry channels at 100 h and 130 h. (C) Representative snapshots of p*Hes7*-Achilles channels in Fire at the start of imaging (130-h post induction). (D) Plots of scaled pHes7-Achilles intensity along time measured in ROIs indicated in (C). All ROIs (top) and single anterior ROI (bottom). The thick red and green lines indicate the mean of intensities from 3 ROIs, and the light pink and grey lines represent the intensity from each single ROI. (E-F) Kymographs of spatial and tempol pHes7-Achilles signals with mCherry-OE (E) and Cdh2-OE iPSM (F). (G) Plots of raw pHes7-Achilles intensity for both –Dox (control) and +Dox (mCherry overexpression in mCherry-OE and Cdh2-p2a-mCherry overexpression in Cdh2-OE). a.u., arbitrary units.

Development • Supplementary information

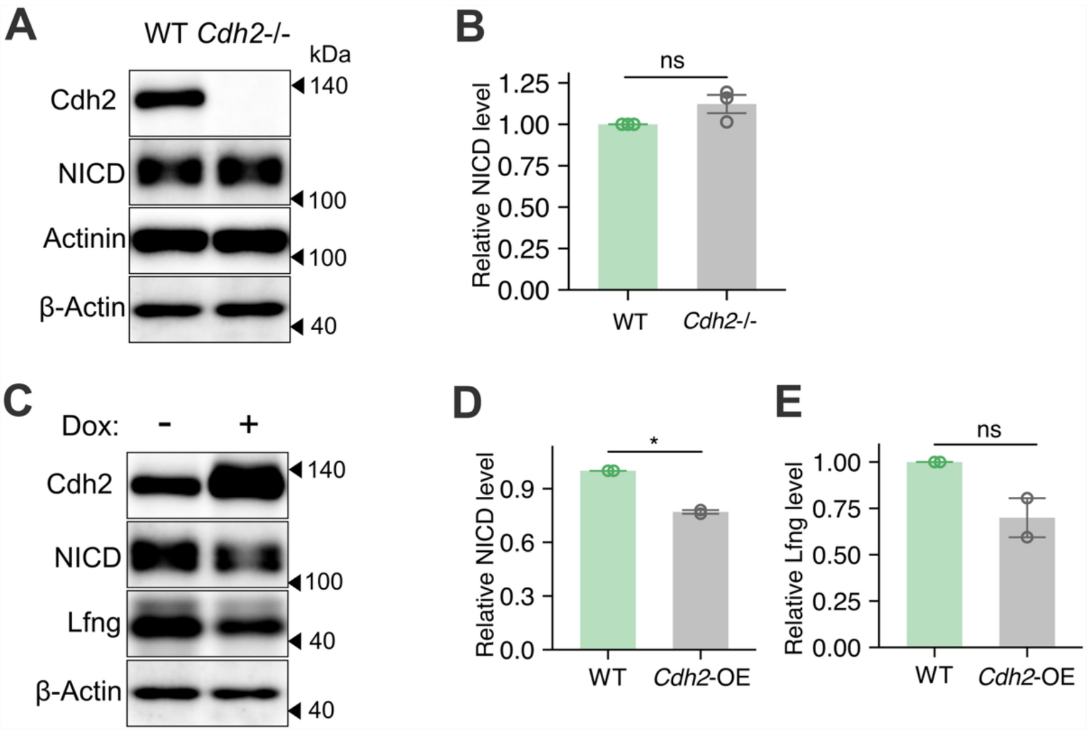

Fig. S7. Effect of Cdh2 on Notch signalling activity in iPSM tissue.
(A, B) NICD levels were quantified by western blotting. WT and *Cdh2*-/- iPSM samples were collected at 96 h. (C-E) NICD and Lfng protein levels were quantified by western blotting. Control (Dox–) and *Cdh2*-overexpressing (OE, Dox+) iPSM tissues were collected at 108 h. Doxycycline was administered at 96 h. Data are shown as the mean ± SEM, t-test: *$p \leq 0.05$, ns: not significant.

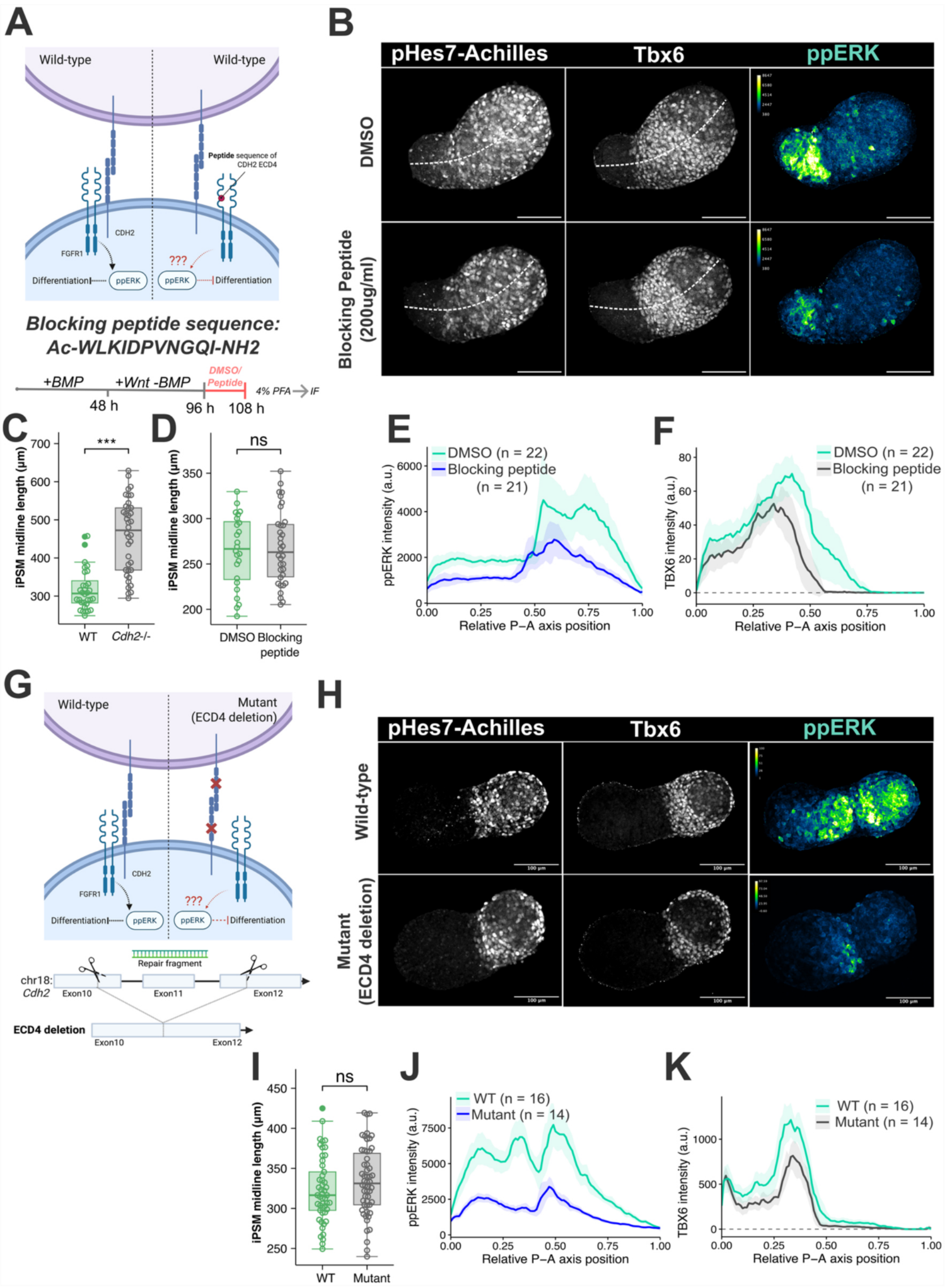

Fig. S8. Effects of interference of Cdh2-FGFR1 interaction on FGF-ppERK activity and PSM cell differentiation.

(A) Illustration of the experimental design for blocking peptide assay. Created in BioRender by Jia, X. (2025). https://BioRender.com/4p2bad5. This figure was sublicensed under CC BY 4.0 terms. (B,E,F) Representative immunofluorescence images (B) and plots comparing ppERK (E) and Tbx6 (F) spatial profiles between applying DMSO and 200 µg/ml blocking peptide, plotted on a normalized major axis (dotted lines in B) where 0 is the posterior end and 1 is the anterior end. Scale bars, 100 µm. a.u., arbitrary units. (C,D) Quantification of iPSM sizes by measuring the midline length along the posterior-anterior axis, mean ± SEM. T-test: ****p*≤ 0.001, ns:

Development • Supplementary information

not significant. (G) Illustration of the experimental design of generating a Cdh2 mutant with only ECD4 removed. To remove the genomic sequence corresponding to amino acid 515-604 in Cdh2 protein, 2 sgRNA (Table S2) for targeting the 5' and 3' regions of deletion were transfected to the pHes7-Achilles mESCs together with a repair fragment designed equivalent to the sequence after removing the targeted region. Created in BioRender by Jia, X. (2025). https://BioRender.com/4qkkg8o. This figure was sublicensed under CC BY 4.0 terms. (H,J,K) Representative immunofluorescence images of ppERK and TBX6 (H) and plots comparing ppERK (J) and TBX6 (K) spatial profiles between wild-type and ECD4 deletion mutant, plotted on a normalized major axis where 0 is the posterior end and 1 is the anterior end. (I) Quantification of iPSM sizes by measuring the midline length along the posterior-anterior axis, mean ± SEM. T-test: ns: not significant.

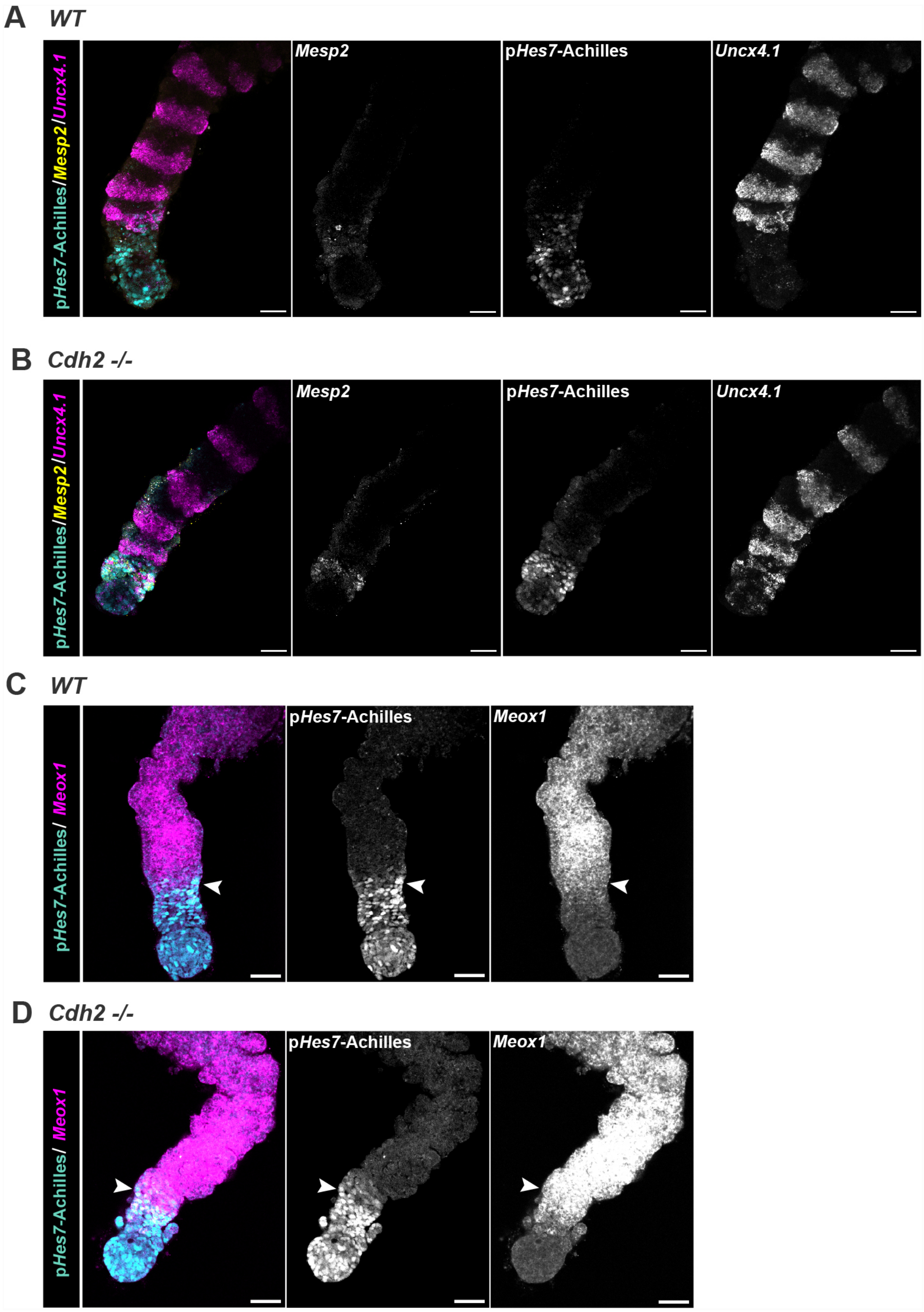

Fig. S9. Hybridization chain reaction staining to examine somite formation. (A,B) iPSM tissue was embedded in 10% Matrigel/ Ndiff227 at 96 h and grown for an additional 20 h before fixation. iPSM tissue was then removed from Matrigel and stained for *Uncx4.1* (magenta) and *Mesp2* (yellow). (C,D) iPSM tissues in the same condition as above are stained for *Meox1* (magenta). Scale bars: 50 µm.

Table S1. Table of the most significant Hes7 binding peaks identified by the ChIP-seq data using anti-Hes7 antibody in E9.5 mouse embryos (see Fig. 1A; Fig. S1 and Methods).

Available for download at
https://journals.biologists.com/dev/article-lookup/doi/10.1242/dev.204743#supplementary-data

Table S2. List of sgRNA and qPCR primer sequencies used in this study

1	sgRNA as negative control	GCGAGGTATTCGGCTCCGCG
2	sgRNA for *Scarf2*-KO	GTCCCAGCCCTCGGGCTCCC
3	sgRNA for *Smoc1*-KO	GTGCAGCTGTCCCCGGCCGG
4	sgRNA for *Lfng*-KO	CAGCCTGGACAGGCCCATCC
5	sgRNA for *Hes7*-KO	GGTGGAACTGACCTGGTCCC
6	sgRNA for *Irf2bp1*-KO	CGACAGTGGCGGTGGCTTGG
7	sgRNA for *Kpnb1*-KO	ACACACTGTGAGGCCGAACT
8	sgRNA for *Tmem131l*-KO	GGGCTCCGGCGCCCGCAGTC
9	sgRNA for *Khdrbs1*-KO	GGAACCCACCACCGTCGCGT
10	sgRNA for *Trim71*-KO	GTTCTTGGGCGGCGGCTCTT
11	sgRNA for *Foxo3*-KO	GAAGGCCCCCCGGCGGCGTG
12	sgRNA for *Jak3*-KO	TGTCATAGTCCTGCGGGCTG
13	sgRNA for *Cdh2*-KO	TCCGCGGCGCTCCCGCTATC
14	sgRNA for *Bcl2l11*-KO	GTTCTGTCTGTAGGGAGGTA
15	sgRNA for *Pcbp1*-KO	GGTGACGGACTGCGGCACGC
16	sgRNA for Ier5-KO	AGGCTCGGGCTCCGGCCAGG
17	sgRNA for *Rab34*-KO	GCCTGCCAGGAGCACCGGAC
18	sgRNA for *Eno3*-KO	GACTCTAGGTCCTGCTCTGC
19	sgRNA for *Tead4*-KO	CCTTGCCACGGAGGAGCGAG
20	sgRNA for *Cited1*-KO	AAGCTTCTGAAGCTGCATGC
21	sgRNA for *Tra2b*-KO	ATTCTCCCATGTCTACTCGA
22	sgRNA for Cdh2-mScarlet KI	ACTGAACGGCAGGACGGACT
23	sgRNA1 for Cdh2 ECD4 deletion	AGCAGTGAGCGTGGTCAGCA
24	sgRNA2 for Cdh2 ECD4 deletion	TATCAATGACAACGCCCCTC
25	qPCR primer1 *Cdh2* Fwd	TGACTGAGGAGCCTATGAAG
26	qPCR primer1 *Cdh2* Rev	TTGTAGAGCTCCACTGTGC
27	qPCR primer2 *Cdh2* Fwd	AGCCTGGAACATATGTGATGA
28	qPCR primer2 *Cdh2* Rev	CCATAAAACGTCATGGCAGTAA
29	qPCR primer *Hes7* Fwd	CGGGAGCGAGCTGAGAATAG
30	qPCR primer *Hes7* Rev	CACGGCGAACTCCAGTATCT
31	qPCR primer *Tbx6* Fwd	ATGTACCATCCACGAGAGTTGT
32	qPCR primer *Tbx6* Rev	GGTAGCGGTAACCCTCTGTC
33	qPCR primer *b-Actin* Fwd	GTGACGTTGACATCCGTAAAGA
34	qPCR primer *b-Actin* Rev	GCCGGACTCATCGTACTCC
35	qPCR primer *Gapdh* Fwd	GCCTTCTCCATGGTGGTGAA
36	qPCR primer *Gapdh* Rev	GCACAGTCAAGGCCGAGAAT

Development • Supplementary information

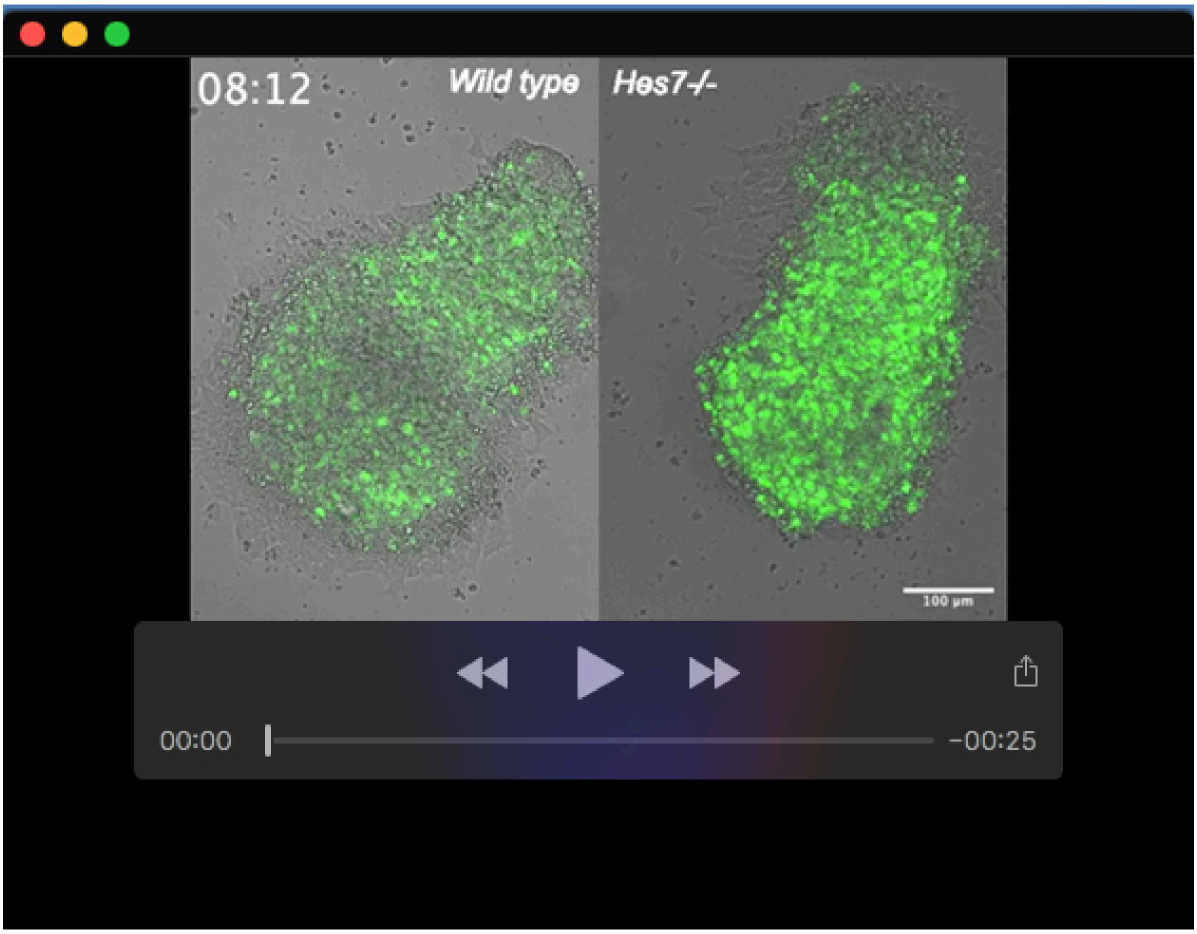

Movie 1. pHes7-Achilles reporter activity in WT (left) and *Hes7*-KO (right) iPSMs, related to Fig.1. Time-lapse live imaging of iPSMs induced from mESCs and plated on the Fibronectin-coated dish in CLBF medium from day 4 to day 6 was performed for WT and mutants in parallel. The images were captured every 3 min for both samples. The bright field and the Achilles channels were merged to present iPSM morphology and pHes7-Achilles oscillations. N=3 independent experiments (rounds of differentiation and live imaging) and n=3 biological replicates (cell lines) for each experiment. Scale bar = 100μm.

Development • Supplementary information

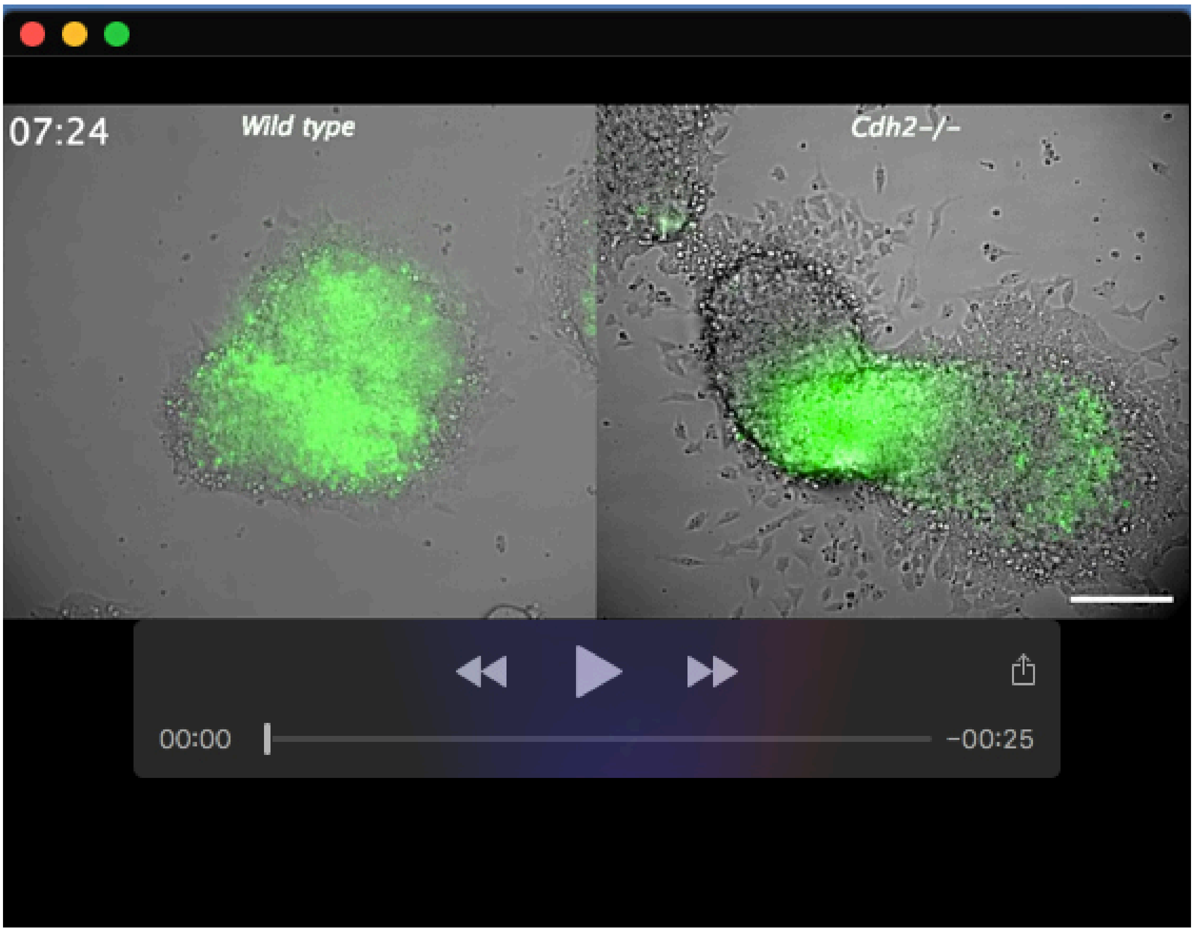

Movie 2. pHes7-Achilles reporter activity in WT (left) and *Cdh2*-KO (right) iPSMs, related to Fig.1. The experimental setup is equivalent to Movie 1. N=3 independent experiments (rounds of differentiation and live imaging) and n=3 biological replicates (cell lines) for each experiment. Scale bar = 100µm.

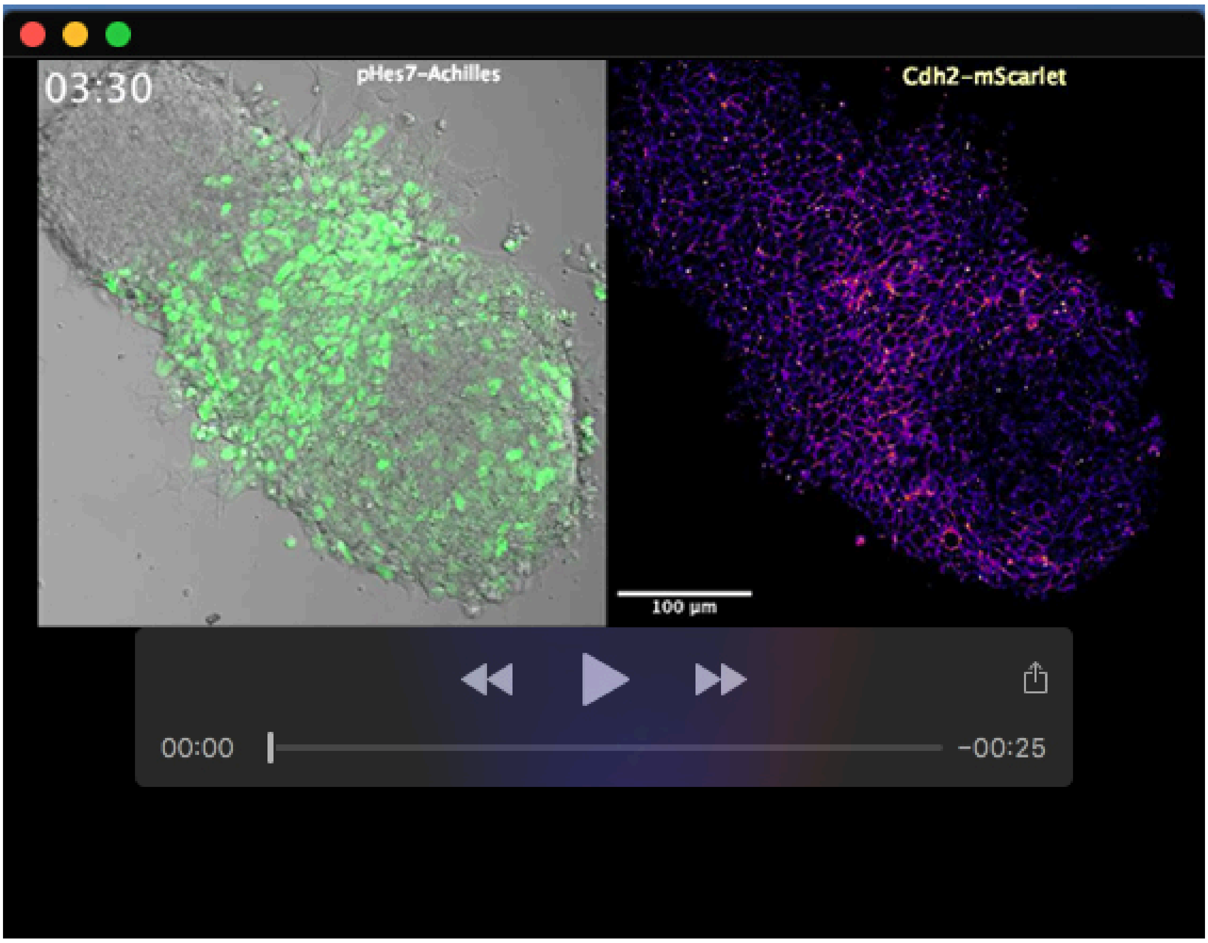

Movie 3. pHes7-Achilles (left) and Cdh2-mScarlet (right) reporter activities in the WT iPSM, related to Fig.3. The experimental setup is similar to Movie 1 with mScarlet channel added. N=2 independent experiments (rounds of differentiation and live imaging) and n=3 biological replicates (cell lines) for each experiment. Scale bar = 100µm.

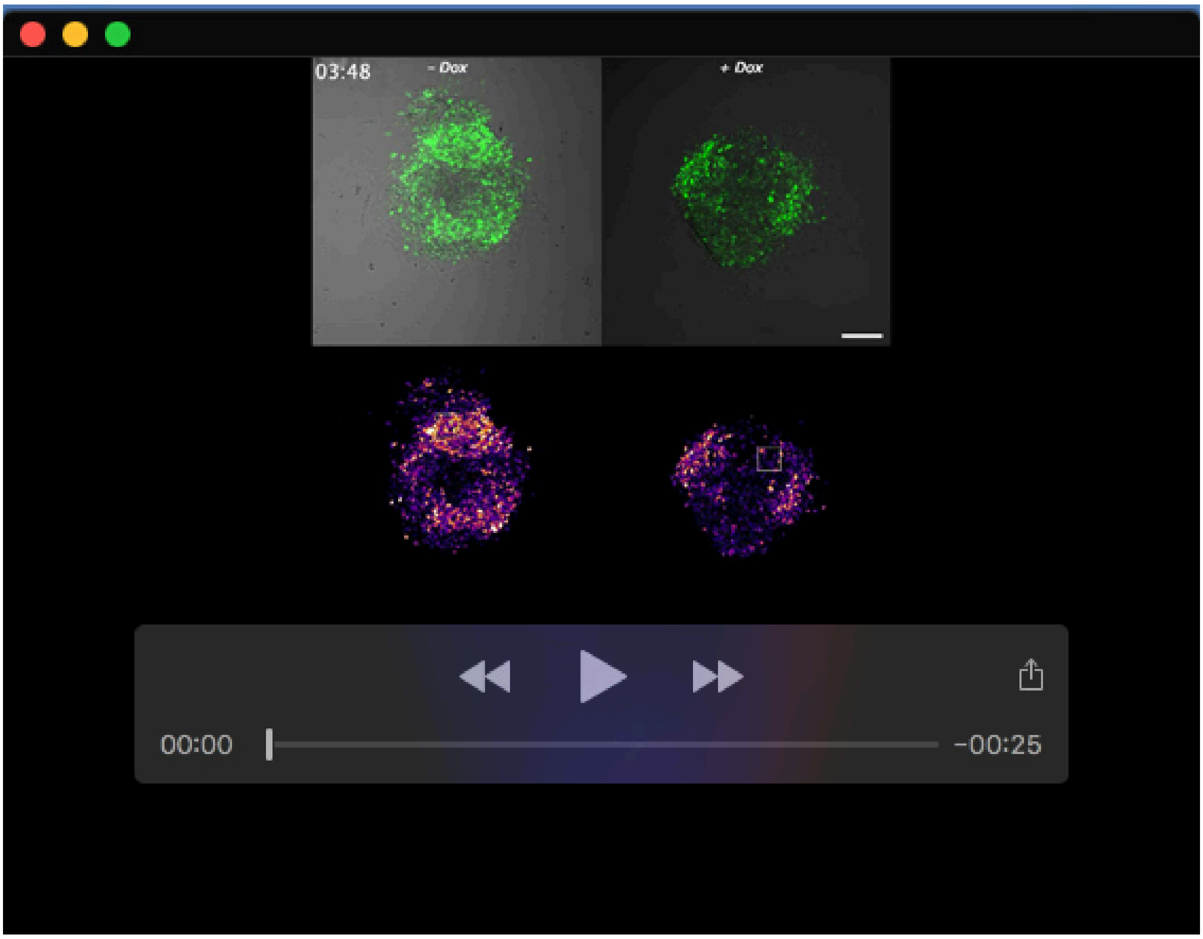

Movie 4. pHes7-Achilles reporter activity in Cdh2-OE iPSMs without (left) and with (right) Dox, related to Fig.4. The experimental setup is similar to Movie 1 with mCherry channel added (Fig. 4B, not shown in the movie). In addition, Dox was added to the medium when transferring iPSMs to the imaging dish. The Achilles channel presented in Fire is supplemented under the merged images for better visualization. The squares indicate the ROIs used in analysis in Fig. 4D. N=3 independent experiments (rounds of differentiation and live imaging) and n=2 biological replicates (cell lines) for each experiment. Scale bar = 100µm.
